# Erratum

**DOI:** 10.1080/17453674.2021.1934801

**Published:** 2021-06-04

Knee flexion contracture impacts functional mobility in children with cerebral palsy with various degree of involvement: a cross-sectional register study of 2,838 individuals

Evelina Hanna Sofia PANTZAR-CASTILLA^1^, Per WRETENBERG^2^, and Jacques RIAD^3^

^1^Department of Orthopedic Surgery, Örebro University Hospital

^2^Department of Orthopedics, Örebro University

^3^Department of Orthopaedics, Skaraborg Hospital, Skövde, Sweden

Correspondence: evelina.pantzar@regionorebrolan.se

Submitted 2020-10-23. Accepted 2021-03-21.

DOI 10.1080/17453674.2021.1912941 Published online ahead of print

Erronous affiliations were published in the online version. The correct affiliations are stated below.

Evelina Hanna Sofia PANTZAR-CASTILLA^1^, Per WRETENBERG^1^, and Jacques RIAD^1,2,3^

^1^Department of Orthopedic Sugery, Örebro University Hospital

^2^Department of Orthopedics, Institute of Clinical Science, Sahlgrenska Academy, University of Gothenburg, Gothenburg, Sweden

^3^Department of Orthopaedics, Skaraborg Hospital, Skövde, Sweden

Correspondence: evelina.pantzar@regionorebrolan.se

Submitted 2020-10-23. Accepted 2021-03-21.

